# Leading Through Noise: Operating Room Noise Challenges for Staff and Leadership Techniques to Ensure Optimal Operational Performance

**DOI:** 10.7759/cureus.69569

**Published:** 2024-09-17

**Authors:** Mena Louis, Nathaniel Grabill, Priscilla Strom, Brian Gibson

**Affiliations:** 1 General Surgery, Northeast Georgia Medical Center Gainesville, Gainesville, USA; 2 Surgery, Northeast Georgia Medical Center Gainesville, Gainesville, USA; 3 Trauma and Acute Care Surgery, Northeast Georgia Medical Center Gainesville, Gainesville, USA

**Keywords:** healthcare team dynamics, noise reduction strategies, operating room noise, or environmental design, or staff well-being, patient safety, stress management in surgery, surgeon leadership, surgical communication protocols, surgical efficiency

## Abstract

Noise and distractions in the operating room (OR) critically impact surgical performance and patient outcomes, particularly in high-stakes environments such as trauma surgery. While historical hospital environments prioritized quiet to facilitate recovery and reduce stress, contemporary ORs, especially those handling trauma cases, face increasing noise challenges due to advanced surgical instruments, alarms, and staff conversations, often surpassing federal exposure limits. This review investigates OR noise sources, including staff activities and equipment, analyzing their effects on cognitive load, communication, and error rates among healthcare workers. It identifies high-risk scenarios and vulnerable groups, highlighting the necessity for targeted interventions.

Key strategies include implementing strict noise control policies, using noise-reducing materials in OR design, and educating staff on noise impacts. Additionally, structured communication protocols and continuous monitoring systems are advocated to enhance operational efficiency and safety. Surgeon leadership is pivotal in balancing assertiveness and empathy to maintain a productive team dynamic. Furthermore, surgeons significantly boost OR efficiency and safety by adopting these protocols, promoting inclusive team dynamics, and applying noise-reduction strategies. These practices safeguard patient care and foster a more collaborative work atmosphere, aligning all team efforts toward optimal patient outcomes. This holistic approach emphasizes the need for continuous improvement and adaptability in surgical practices to meet modern healthcare demands, particularly in trauma surgery's fast-paced, unpredictable realm. Collectively, these measures can enhance patient safety and improve conditions for surgical teams, providing a framework for quieter, more focused OR environments that ultimately elevate surgical outcomes and healthcare quality.

## Introduction and background

In the past, hospitals were sanctuaries of silence, with strict noise control measures both inside and outside the facilities [1,2]. Uniformed nurses enforced quietness within, while external signs declared “Hospital-Quiet Zone” to maintain a serene environment conducive to patient recovery [1,2]. However, over the past few decades, the atmosphere within hospitals, particularly in operating rooms (ORs), has transformed significantly [3,4]. Modern ORs are now filled with the sounds of advanced medical equipment, alarms, and often, the chatter of medical staff [5]. This shift has brought new challenges for maintaining a focused and safe environment for patients and healthcare professionals [6,7].

Noise levels in ORs frequently exceed those of a busy highway, reaching peaks of up to 120 dBA during surgical procedures, far surpassing federal limits for occupational noise exposure [1,8​]. The introduction of high-powered surgical tools, constant alarms, and even background music has contributed to an environment that can be as noisy as a cafeteria at noon [8-10]. Such high noise levels hinder effective communication among the surgical team and increase cognitive load and stress, potentially leading to errors and compromised patient safety [5,8,11,12].

Crowdedness and side conversations further exacerbate these challenges [8,13,14]. The presence of unnecessary personnel and equipment in the OR can disrupt the workflow, leading to delays and distractions [8,15,16]. Conversations unrelated to the surgical procedure add to the overall noise level, diverting attention from critical tasks and increasing the risk of miscommunication [8,17,18]. Studies have shown that such distractions are linked to poorer performance and higher rates of surgical errors, directly impacting patient morbidity and outcomes [8,19,20].

Addressing these issues requires a comprehensive approach, combining strict noise control policies, environmental design modifications, and structured communication protocols [8,16,21]. By implementing these measures, hospitals can create a quieter, more focused environment in the OR, enhancing patient safety and surgical team working conditions [12,22]. This article explores the sources of noise and distractions in the OR, their impact on healthcare professionals and patient outcomes, and practical solutions to mitigate these risks, aiming to improve overall healthcare quality and safety [8,23,24].

## Review

Historical noise levels in hospitals and ORs

In the 1960s, hospitals were meticulously designed as quiet sanctuaries, strongly emphasizing controlling noise to create environments that fostered patient recovery and enhanced healthcare efficiency. This era saw the implementation of design philosophies and architectural practices focused on minimizing ambient noise and recognizing the correlation between a serene atmosphere and faster patient recovery rates [25]. Uniformed nurses were crucial in maintaining these quiet zones, actively monitoring and managing noise levels within hospital corridors and wards [7,26]. Their efforts were supported by visible external signs that designated quiet areas, reinforcing the hospital's commitment to tranquility [27]. This proactive approach to noise management was more than a matter of comfort. Still, it was deeply rooted in emerging research, which suggested that reduced noise levels could significantly decrease patient stress and improve overall healthcare outcomes [28]. The American College of Surgeons (ACS) highlighted these measures as essential, emphasizing their critical role in reducing environmental stressors that could impede recovery and compromise patient care [28]. By creating these noise-controlled environments, hospitals from this period set a precedent for considering acoustic management as integral to hospital design and patient care strategies. This practice would shape healthcare facilities for decades [25,9].

Early Reports of Increasing Noise

The early 1970s marked a pivotal moment in the history of OR environments as a significant shift in noise levels was first documented. Shapiro and Berland's seminal study in 1972 provided concrete evidence, showing that noise levels in ORs often surpassed those found on busy highways, peaking at as high as 85 dBA during routine surgical procedures [18]. This alarming comparison showed the stark contrast between the controlled quiet of the 1960s and the emerging acoustic landscape of modern medical facilities [26]. The rise in noise was primarily attributed to the increasing adoption of advanced surgical instruments and technologies [27]. As these tools became integral to contemporary medical practices, their operational sounds contributed substantially to hospital ambient noise [28]. This period did not just signify a rise in decibels but marked the beginning of a trend where technological advancements, improving surgical outcomes and capabilities, inadvertently introduced a new challenge of managing noise pollution within healthcare settings [29]. The integration of electronic devices, from monitors to complex imaging machines, each added layers of auditory output that, when combined, created a cacophony that could potentially hinder both staff performance and patient recovery [30]. This early documentation spurred further research and discussions about new strategies to mitigate noise in hospitals, setting the stage for ongoing efforts to balance technological benefits with environmental control.

Progression through the decades

As healthcare technology evolved over the subsequent decades, so too did the acoustic challenges within ORs [31]. The trend of increasing noise levels, which began to be noticeably documented in the early 1970s, continued to escalate into the late 20th and early 21st centuries [32]. By the 1990s, the situation had intensified significantly, with Hodge and Thompson reporting that intermittent sounds reached up to 108 dBA during major surgical procedures [14]. This level of noise not only surpassed earlier records but also began to encroach upon thresholds typically associated with heavy industry rather than medical environments [14,33].

The introduction and widespread adoption of more complex and powerful surgical tools contributed substantially to this increase [34]. For instance, high-speed drills, electrocautery devices, and automated suction equipment became staples in surgeries (Table 1), each emitting levels of noise that cumulatively challenged the auditory environment of the OR [35].

**Table 1:** Noise Levels of Common Surgical Power Tools The noise levels indicated are approximate ranges based on typical usage during surgical procedures. Actual noise levels can vary depending on specific operating conditions and tool settings [18,36]. dBA: Decibels A-weighted. This is a unit of sound measurement that approximates the sensitivity of the human ear to different frequencies of sound. Higher dBA values indicate louder sound levels.

Surgical Tool	Type	Noise Level (dBA)
High-Speed Drill	Orthopedic/Neurosurgery	95-110 dBA
Electrocautery Device	General Surgery	70-90 dBA
Automated Suction Equipment	General Surgery	85-95 dBA
Bone Saw	Orthopedic Surgery	110-120 dBA
Surgical Aspirator	Various Procedures	75-85 dBA
Surgical Laser	Ophthalmic/ENT Surgery	60-80 dBA
Ultrasonic Scalpel	Laparoscopic Surgery	70-85 dBA

By 2012, the scenario had advanced further. Fritsch et al. observed noise levels peaking at an unprecedented 131 dBA during simulated otolaryngological surgery [36]. This finding was particularly significant as it shows the compounded impact of both the variety and volume of modern surgical tools and equipment in use. Such high decibel levels, comparable to those experienced at a rock concert or a jet takeoff, highlighted an urgent need for revisiting and revising strategies to manage noise in ORs (Table 2). The escalating noise levels not only posed risks to patient safety due to potential communication barriers but also raised serious concerns about the long-term hearing health and overall well-being of medical staff [37].

**Table 2:** Noise Levels During Various Surgical Procedures The noise levels indicated are representative of typical procedures, with variations based on equipment used, surgical techniques, and the duration of the procedure [18,21]. dBA: Decibels A-weighted. The A-weighted decibel scale accounts for the varying sensitivity of human hearing at different frequencies, making it suitable for measuring the noise levels experienced by surgical staff and patients during procedures.

Surgical Procedure	Typical Noise Level (dBA)
Orthopedic Surgery (e.g., joint replacement)	100-120 dBA
Neurosurgery	95-110 dBA
General Surgery (e.g., laparotomy)	85-100 dBA
Otolaryngeal Surgery (e.g., sinus surgery)	100-131 dBA
Cardiothoracic Surgery	90-110 dBA
Laparoscopic Surgery	85-95 dBA
Dental Surgery	80-90 dBA

Tables 1-2 represent the noise levels of various surgical tools and procedures. High noise levels, particularly those exceeding 100 dBA, can pose significant risks to both healthcare workers and patients, including communication difficulties, increased stress, and potential hearing damage for OR staff. This ongoing rise in decibel levels in ORs reflects the complex interplay between technological advancement and environmental control, prompting a reevaluation of how surgical environments are designed, from acoustic paneling to the strategic placement and use of equipment [38].

Modern noise levels

Current Noise Levels in ORs

Recent studies continue to document alarmingly high noise levels in modern ORs, affirming ongoing concerns about auditory conditions in these critical settings [39]. The research highlighted by a comprehensive review from PSNet confirms that the average noise levels in ORs routinely surpass the 85 dBA threshold set by the Occupational Safety and Health Administration (OSHA) for occupational noise exposure [40-42]. In many instances, noise levels during specific surgical procedures can escalate to as high as 120 dBA, reflecting the intensity and frequency of sounds generated in the OR [43]. This persistent issue is largely due to the integration of advanced surgical instruments, the continuous operation of multiple alarms from various monitoring devices, and the general hum of background conversations and music played during operations [44,45]. Each of these factors contributes to a complex auditory environment where noise accumulates, compounding the challenge of maintaining a conducive surgical setting [30].

Comparison to Federal Limits

The ramifications of such high noise levels in ORs are profound, especially when considering the established federal limits. The frequent exceedance of these safety thresholds highlights a critical health concern for OR personnel. Prolonged exposure to noise levels above recommended limits can have detrimental effects on the auditory health of medical staff, potentially leading to permanent hearing loss, increased psychological stress, and overall burnout. The urgency to implement effective noise control measures is underscored by these findings, emphasizing the need for healthcare facilities to adopt more stringent noise reduction protocols and acoustic management strategies to safeguard staff well-being and enhance the operational quality of surgical practices [26,27,31]. These insights call for a strategic reevaluation of how ORs are equipped and managed, suggesting that more attention be given to acoustic design and the adoption of quieter surgical technologies.

Impact on healthcare workers and patients

Effects on Performance and Stress Levels

High noise levels in the OR have a profound impact on the performance and well-being of healthcare workers. The ACS points out that such environmental stress can disrupt essential communication, significantly increase cognitive load, and induce stress among the surgical team. These conditions not only heighten the likelihood of errors but also reduce overall efficiency during surgical procedures. Prolonged exposure to high-decibel environments can lead to fatigue, reduced concentration, and impaired decision-making, which are critical factors in high-stakes medical settings where precision and attention to detail are paramount [7,23,32].

Health Consequences

Continuous exposure to elevated noise levels is associated with several chronic health issues for OR staff. Studies, including research by Wallace et al., have shown that a significant percentage of anesthesiologists display signs of hearing impairment, such as abnormal audiograms, especially those under the age of 55, indicating that occupational noise exposure in the OR can lead to more severe hearing loss than that found in the general population. Furthermore, noise acts as a biological stressor, which has been linked to increased heart rate and blood pressure, escalating the risk of developing cardiovascular diseases over time [24,25,27,30,31].

Patient Safety and Outcomes

The safety and outcomes of patients are directly impacted by the noise levels in the OR. High ambient noise can disrupt the surgical team's focus and communication, leading to procedural errors and potential complications that adversely affect patient outcomes. Such distractions can be particularly detrimental during critical phases of surgery where the precision of actions and decisions is crucial. Moreover, noise can also interfere with the effective operation of monitoring devices, which are vital for maintaining patient safety during procedures [4,5,7,33].

Recovery Times and Overall Safety

Patients are especially susceptible to the adverse effects of noise, which can induce stress and discomfort, potentially complicating their recovery process. Elevated noise levels during post-operative care can disturb patients' rest and delay healing, leading to extended hospital stays and increased healthcare costs. Furthermore, the interference caused by noise with monitoring devices can lead to delayed responses to critical patient alarms, further compromising patient safety and care quality during recovery [16,31,34,35]. The cumulative effect of noise-induced stress and cognitive overload can significantly deteriorate the quality of care provided, emphasizing the need for effective noise management strategies in surgical environments.

Discussion

The literature on OR environments robustly indicates that increasing noise levels correlate with diminished surgical performance and patient safety [21]. This review synthesizes historical data and contemporary studies to argue that systemic changes are necessary to mitigate these effects [17]. Historically, ORs were maintained as quiet zones to facilitate concentration and minimize stress, both for the surgical team and the patient [1,6]. However, advancements in surgical technology and increased OR activity have led to noise levels that often exceed those deemed safe by federal regulations [12]. Current data demonstrates that noise levels during various surgical procedures can reach as high as 120 dBA, well over the 85 dBA limit set by OSHA, underscoring a persistent and escalating challenge [3,20].

For patients, the stakes are similarly high. Noise not only disrupts procedural efficacy but also compromises patient safety [14]. High noise levels are associated with increased surgical complications, such as infections and prolonged recovery times, due to direct and indirect noise impacts [19]. Direct impacts include interference with surgical focus and procedural execution, while indirect impacts involve the impairment of alarm systems and communication necessary for patient monitoring [36]. Addressing these challenges requires a multifaceted approach, particularly in the role of surgeons who must lead by example to foster a cooperative and respectful OR environment [37,38]. Surgeons are pivotal in setting the tone for communication and behavior in the OR [29]. They must balance assertiveness with approachability to effectively lead without being perceived as rude or dismissive [13,37]. This balance is crucial because the primary goal of OR staff is to function as a unified team dedicated to patient safety and care [39].

Effective leadership involves clear and respectful communication, regular feedback, and acknowledging team members' contributions. Surgeons can facilitate this by:

Implementing Structured Communication Protocols

Adopting tools like checklists and briefings can help streamline communication and ensure that all team members are on the same page without the need for repeated interruptions or loud conversations [40].

Encouraging a Team-Oriented Atmosphere

Promoting an environment where each member feels valued and heard can reduce perceived rudeness or dismissiveness. This includes involving staff in decision-making processes and openly discussing strategies for noise reduction and patient safety [41].

Training on Interpersonal Skills

Surgeons can benefit from training that enhances their ability to deliver constructive criticism, manage conflicts, and communicate effectively under stress, ensuring that their leadership style is both respected and collaborative [38,39].

Regularly Reviewing Team Dynamics

Continuous assessment of team dynamics and noise levels in the OR can help identify areas for improvement, allowing adjustments that foster a better working environment that enhances patient care [42]. By implementing these strategies, surgeons can lead their teams more effectively, ensuring the OR remains a high-efficiency and safety zone conducive to optimal patient recovery and a supportive work environment for healthcare professionals [16,39]. Effective leadership in the OR requires a delicate balance between maintaining authority and fostering a collaborative atmosphere [39]. Surgeons need to communicate in ways that are perceived as constructive rather than critical, emphasizing teamwork and mutual respect [22,42].

To avoid being seen as rude or dismissive, surgeons can focus on inclusive communication techniques, such as regularly soliciting input from all team members and acknowledging the diverse contributions of the surgical team [22,39,42]. This approach improves team dynamics and enhances the collective focus on patient safety [37]. Surgeons should also practice active listening skills, demonstrating attentiveness to the concerns and suggestions of OR staff, which can significantly reduce perceptions of dismissiveness [43]. By reinforcing positive interactions and openly appreciating each team member's role, surgeons strengthen team morale and promote a culture of respect and cooperation. Regular interpersonal communication and conflict resolution training can further enhance these skills [44]. These sessions can provide surgeons and OR staff with the tools needed to navigate high-stress situations effectively, ensuring clear and respectful interactions even under pressure [21].

While many noise-generating factors in the OR, such as the use of high-speed surgical tools and essential monitoring equipment, are inherently difficult to control, surgeons as leaders can significantly impact the overall noise environment by focusing on modifiable factors. For instance, surgeons can enforce strict protocols that minimize unnecessary conversations during critical phases of surgery and ensure that only essential personnel are present in the OR, thereby reducing ambient noise levels. Additionally, they can advocate for the use of newer, quieter surgical instruments and regularly scheduled maintenance to prevent excess noise from aging equipment. By prioritizing these controllable elements, surgeons can create a more focused and efficient OR environment, even amidst the unavoidable noise generated by necessary equipment. This proactive approach in leadership not only mitigates some of the acoustic challenges but also reinforces the importance of maintaining a controlled and patient-centered surgical environment. Ultimately, the goal is to create an environment where the surgical team operates seamlessly as a unit, with each member feeling valued and heard [16]. This improves the work atmosphere and directly contributes to the overall success of surgical outcomes and patient satisfaction [27].

## Conclusions

Noise and distractions in the OR can negatively affect surgical performance and patient safety. Surgeons, as leaders, play a key role in mitigating these issues by enforcing structured communication protocols and fostering effective teamwork. Implementing noise-reduction strategies improves both safety and efficiency in the OR. Adapting these practices is essential to meet the ongoing demands of modern healthcare.
